# Mercury in Children: Current State on Exposure through Human Biomonitoring Studies

**DOI:** 10.3390/ijerph14050519

**Published:** 2017-05-12

**Authors:** Flavia Ruggieri, Costanza Majorani, Francesco Domanico, Alessandro Alimonti

**Affiliations:** Department of Environment and Health, Istituto Superiore di Sanità, Viale Regina Elena 299, 00161 Rome, Italy; flavia.ruggieri@iss.it (F.R.); costanza.majorani@iss.it (C.M.); giudoma@tin.it (F.D.)

**Keywords:** mercury exposure, biomarkers, human biomonitoring (HBM), children’s health

## Abstract

Mercury (Hg) in children has multiple exposure sources and the toxicity of Hg compounds depends on exposure routes, dose, timing of exposure, and developmental stage (be it prenatal or postnatal). Over the last decades, Hg was widely recognized as a threat to the children’s health and there have been acknowledgements at the international level of the need of a global policy intervention—like the Minamata treaty—aimed at reducing or preventing Hg exposure and protecting the child health. National human biomonitoring (HBM) data has demonstrated that low levels of exposure of Hg are still an important health concern for children, which no one country can solve alone. Although independent HBM surveys have provided the basis for the achievements of exposure mitigation in specific contexts, a new paradigm for a coordinated global monitoring of children’s exposure, aimed at a reliable decision-making tool at global level is yet a great challenge for the next future. The objective of the present review is to describe current HBM studies on Hg exposure in children, taking into account the potential pathways of Hg exposure and the actual Hg exposure levels assessed by different biomarkers.

## 1. Background

Children are considered especially vulnerable to environmental threats since when exposed to stressors they respond differently than adults [1], mostly due to their immature immune defenses. In addition, the active time spent outdoor and specific behaviors (such as frequent hand-to-mouth activity and play on and crawl across the floor), increase the exposure risk in children. Also in the womb, the child can be exposed to adverse environmental risk factors that may imply various diseases later in life [2]. The specific and continuous growth originates a unique susceptibility of children observed through critical time-windows not seen in adults. Over the last decades, some especially long-lasting contaminants, like mercury (Hg), were widely recognized as a threat to the children’s health, and the need to protect the environment in order to safeguard the child’s health has been broadly accepted at the international level [3].

Mercury is ubiquitous in the global environment and occurs both from anthropogenic and natural sources. It is ranked third of the most toxic elements to human health by the United States (US) Government Agency for Toxic Substances and Disease Registry (ATSDR); it exists in various forms: organic (e.g., methyl- and ethyl-mercury), inorganic (e.g., mercuric chloride), and elemental (or metallic) [4]. Each of these forms has a species-specific toxicity that involves different impacts on health surveillance and, then, different countermeasures to avoid exposure. Mercury has multiple exposure sources (e.g., use of Hg-containing skin creams and soaps, fish consumption by themselves or by pregnant women, use of pediatric vaccines, etc.) and the toxicity of Hg compounds depends on the exposure pathway (ingestion, inhalation, transdermal, and transplacental absorption), dose, timing of exposure (acute, chronic), developmental phase, being prenatal or postnatal (fetal, infants, children and adolescent) stage. However, exposure to different Hg compounds may occur contemporaneously—often with other neurotoxic substances—acting together as a threat to the development of the child [5]. More than one system can be affected and different pathology may arise. The main concern is related to the development of the central nervous system (CNS) because mercury’s neurotoxic effects can be a consequence of prenatal or postnatal exposure [6,7]. Traditionally, it has been assumed that fish consumption is the most important pathway of exposure in children, and that methylmercury (MeHg) is the most important hazardous compound. Major events and studies about recognition of MeHg toxicity in children are listed in Table 1.

The first report on MeHg neurotoxicity in infants was recorded in 1952 by Engleson and Herner [8]. In 1956, a seafood related disease causing several neurological symptoms and birth defects was discovered in Minamata Bay in Japan, but only in 1968, was MeHg acknowledged by the governmental authorities to be the cause of Minamata disease [9]. In the early 1970s, an outbreak occurred in Iraq caused by wheat seeds covered in a MeHg-derived fungicide, and 459 deaths of adults were directly related to the MeHg poisoning [10]. In the same period, Spiker et al. [11] reported the first experimental study on the delayed effects of developmental neurotoxicity in rats exposed to MeHg, hence, great attention was paid to the risk of fetal and childhood exposure. Harada [12] confirmed that infants, exposed through their mother, presented cerebral palsy-like symptoms, even when their mother had displayed moderate or no manifestation of poisoning [12]. A few years later, Marsh et al. [13] and Harada [14] observed mental retardation, cerebellar ataxia, primitive reflexes, dysarthria, and hyperkinesia in the same scenarios. The first epidemiological report on adverse effects in children, related to maternal fish and shellfish intake during pregnancy, was documented in New Zealand in 1986 [15], and confirmed ten years later by a prospective study in the Faroe Islands on the adverse effects of maternal seafood intake on children’s health [16,17,18]. This last cohort study, together with the study conducted on children of the Seychelles Islands, are worthy of particular mention [19,20,21,22,23,24,25,26,27,28]; they are the two major longitudinal cohort studies conducted to date in which children were meticulously followed through adolescence to assess the different neuropsychological performances as a consequence of current and past exposure. The Faroes study observed that even a low exposure to MeHg during the early developmental stages could cause neurobehavioral deficits later in life, including problems on several neurophysiological domains like memory and attention, language, visual-spatial and motor skills [17]. No effects were, initially, observed in the Seychelles cohort [20,23,28]. Successively, a more recent study on the same Seychelles cohort verified the occurrence of an association between adverse symptoms on higher-order cognitive functions (e.g., reductions of motor skill) and higher fetal exposure to MeHg [26,27].

In contrast to MeHg, little is known about the neurotoxicity caused by elemental Hg (Hg^0^ that exists as liquid metallic form or vapor) and inorganic Hg compounds (mainly mercuric mercury Hg^2+^ like mercuric chloride) on fetus and child development. However, it is well established that all forms of Hg are toxic [29]. In the last years, several studies conducted in European countries [30,31,32,33,34,35], USA [36], Canada [37], Japan [38], and China [39] have demonstrated that exposure to Hg is still a crucial public health concern, which no one country can solve alone. In 2013, global measures were adopted by the Minamata Convention aiming to reduce and eliminate sources of exposure to Hg (http://www.mercuryconvention.org). In 2015, the Convention was signed by more than half of the Member States of the World Health Organization (WHO) European Region during a health-focused meeting. Currently, a deep collaboration between the WHO European Center for Environment and Health (WHO ECEH) and the United Nations Environment Programme (UNEP) is being improved, aiming to plan a human biomonitoring (HBM) at global level as a key tool to evaluate the baseline condition and the capacity of the convention to decrease human exposures in children and adults [40].

This review builds on existing literature, highlighting current understanding about children’s exposure to all forms of Hg derived by HBM studies, aiming to identify research gaps on health surveillance, and concluding with what we consider unresolved issues and efforts needed to resolve them.

## 2. Sources of Exposure

Children face exposure risks to all forms of Hg from numerous different sources and routes of exposure; above all, in developing nations, particular exposure risks are related to religious and cultural practices, occupational activities such as gold mining extraction, as well as survival diet exclusively based on fish consumption. Hg exposures are not equally distributed in the world, due to a large variability of Hg deposition. Therefore, geographical features can influence the environmental Hg, and the resulting large variability of environmentally mediated exposures makes it difficult to develop successful strategies able to protect children in specific local communities and regions [29].

Generally, childhood Hg exposures begin at the point of conception, and beyond the critical time of gestation, it continues throughout the stages of infancy, childhood, and adolescence. During prenatal exposure, the sources of exposure for pregnant women are also sources of fetal exposure, and, among various factors, special emphasis has been addressed to mother’s dietary intake of fish, shell-fish or marine mammals (the last one, particularly, in the Arctic and sub-Arctic populations). Differently, in some regions of the world such as China, MeHg exposure via a rice-based diet is an increasing risk factor [29]. A pathway of concern for pregnant women is also represented by Hg vapors released from maternal dental amalgams, which may contain up to 50% of elemental Hg [41], and, particularly among women living in developing nations, by Hg vapors released during mining activity. During pregnancy, maternal exposure to Hg could produce damage on neurodevelopmental systems such as behavioral, cognitive patterns and motor skills, and on the immune and reproduction systems, noticeable later in life [7]. For this reason, the quality of life in adolescents and adults may be affected by the persistence of prenatal exposure to Hg.

A highly efficient gastrointestinal absorption, physiological immaturity of homeostasis and detoxification mechanisms could be the reasons why the infants are at higher risk than older children and adults. Breast milk consumption is the main pathway of exposure in infants, but also the use of specific products such as teething powders, soaps, and organomercurial compounds may represent sources of exposure [29]. Both organic and inorganic Hg occur in breast milk, but the mammary gland physiology causes a preferential enrichment of inorganic Hg, as the latter rapidly enters the plasma and, therefore, the breast milk. This is supported by the preferentially partition of MeHg to erythrocytes [42].

To support their growth, children consume more food on a broad weight basis than adults do. For example, data from the National Health and Nutrition Examination Survey (NHANES) in 1999–2000 showed that children aged <10 years were exposed to about 0.33 µg of Hg per kilogram of body weight per day (0.33 µg/kg bw/day) in the food they eat, while children aged 11–14 years presented a Hg dose (0.15 µg/kg bw/day), more comparable to that seen in adults (0.05 µg/kg bw/day) than to that of younger children [36]. As a result, the potential harmful exposure in children exceeds those of most adults [29]. In developed countries, unintentional accidents (e.g., from broken thermometers, fluorescent light bulbs and other pressure gauges or liquid metal used in school laboratories), or specific products (e.g., mercury-containing paints) represent the main routes of children’s exposure to elemental Hg, while exposure to inorganic Hg occurs mainly through the use of cosmetics (e.g., skin-lightening creams and soaps) containing Hg salts [29]. Although children are not typically exposed to Hg in active workplaces, some former industrial facilities that used Hg (subsequently converted to residences or childcare facilities), or take-home exposure from occupationally exposed parents can lead to significant elemental Hg exposure [43]. Furthermore, organic Hg intoxication in children may result from diet and vaccines. Ethyl mercury (EtHg) has been used as a topical antiseptic and as antifungal agent in multi-dose vaccine vials routinely given to children in the form of thimerosal (which contains 49.6% of EtHg by weight) [44]. For this reason, in contrast to some developing countries, thimerosal has been banned from most vaccines in the United States. Except for the wealthier countries of the European Union (EU), thimerosal (multi-dose vials) is still prescribed to pregnant mothers and infants worldwide. At the EU level, the lack of precise regulations gives the basis of an increased concern, since the harmful effects of high dose EtHg seem to be close to those of MeHg.

A broad range of adverse effects on endocrine systems by means of specific cytotoxicity in tissues, variations in hormone concentrations, or perturbations of the steroidogenesis pathway, were observed during puberty [7,36]. Moreover, due to development of the nervous system during the adolescence, several adverse neurological effects were displayed in older children [7,45].

## 3. Biomarkers of Exposure

Once a chemical enters the body by different routes of exposure, it is generally distributed by the blood stream and released as its parent compound or its metabolites, and/or stored in tissues/organs. The physicochemical properties of various Hg species determine their metabolism and excretion routes, and the knowledge of these pathways is crucial to identify an appropriate matrix for monitoring the Hg body burden. An overview of the toxicological features of each Hg species (e.g., sources of exposure, toxicokinetics, distribution and biotransformation, excretion, and critical target organs) is displayed in Table 2.

A biomarker of exposure should have the ability to define the occurrence and extent of the total body burden by the measurement of a chemical (parent compound or metabolite) in a specific biological matrix. The selection of a reliable biomarker of exposure, as a biomonitoring tool, is a crucial key for: (i) monitoring actual exposures; (ii) identifying the extent to which the perturbation of homeostatic pathways can lead to the development of adverse health outcomes; (iii) assessing precise risk distribution in the population, and (iv) supporting public health protection policies focused on exposure prevention. The usefulness of a biomarker of exposure should be determined by using the following criteria: (i) how well the biomarker of internal dose—hair, blood, cord-blood, urine, saliva, breast milk, and/or other specimens, such as feces, teeth, and nails—is a real measure of external exposure; (ii) how well the biomarker is an indication of the true extent of exposure at the target site; and (iii) how well the time-variable concentration profile of the biomarker reproduces the temporal patterns of the effective dose within the target site [29].

Moreover, the selection of a suitable biomarker should also take into account other criteria such as availability of health-based guidance values and analytical methods with adequate performances (i.e., limits of detection), complexity of the fieldwork procedures and costs. The last two items may be crucial, above all, when the HBM study is conducted in terms of cohorts (i.e., large number of individuals and specimens) comprising the evaluation of the effectiveness of the regulatory interventions to reduce and prevent exposure. Discussion on each biomarker used for assessing different Hg compound exposures in children is reported below.

### 3.1. Methylmercury

**Figure 1:**
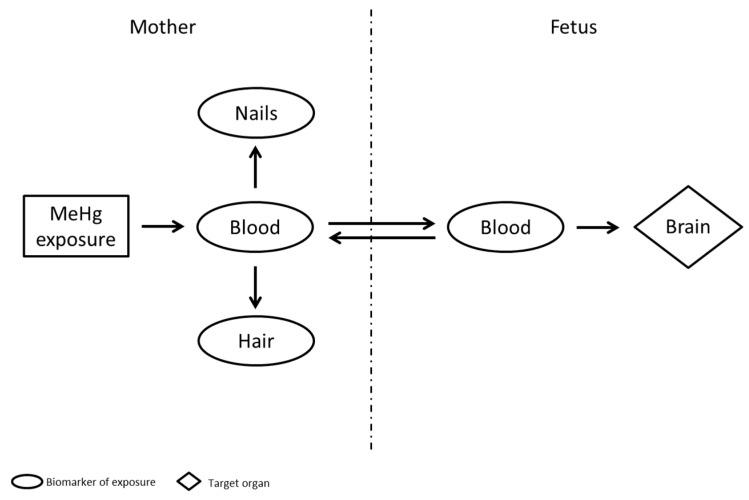
Relationship among the various biomarkers used for characterizing methylmercury (MeHg) exposure.

The target organ of MeHg exposure during gestation is the fetal brain; therefore, a reliable biomarker for assessing MeHg exposure in the fetus must be able to predict the effects on the development of the child. The relationship among the various biomarkers used to assess MeHg exposure is shown in Figure 1.

The concentration of Hg quantified in different biological compartments (i.e., maternal blood, fetal/cord blood, maternal hair, and maternal nails) may be considered as a biomarker of exposure, nevertheless, each matrix provides different exposure information. The specific information and strength or weaknesses on each biomarker are summarized in Table 3.

In populations with high fish consumption the measurement of total Hg in maternal blood is generally suitable for the assessment of the current internal exposure to MeHg, because after absorption, it crosses the placental membranes and, then, it is distributed to the fetal brain [4]. Data obtained from children (aged 1–6 years) and women over the NHANES in 1999–2000, highlighted that when the total Hg exceeded 4 μg/L, more than 90% of occurring Hg was MeHg [36]. Hematocrit affects whole blood Hg concentrations, thus, some researchers used the total Hg in Red Blood Cells (RBCs)—after separation from plasma/serum which contains inorganic Hg—as a more accurate measure of MeHg [47]. The mean half-life of MeHg in human blood is about 50 days, therefore maternal blood Hg concentration reflects short-term exposure [46]. However, for mothers with a regular pattern of fish consumption, a steady-state blood Hg concentrations is achieved (i.e., daily Hg removal from blood is the same to the daily Hg intake) and, under such conditions, an individual spot sample provide a good estimation of the concentration over time. Compared to maternal blood, the advantage of cord blood is that it circulates in the fetal body and represents a better measure of the MeHg levels in fetal target organs, including the fetal brain [46]. Several studies observed a higher Hg concentration in cord blood than in the corresponding maternal blood (30–70%), likely due to the easy transfer of MeHg through the placenta, the greater affinity of MeHg to fetal hemoglobin, and the higher hematocrit in newborns compared to their mothers [38,47,48,49]. Since the fetus has not independent mechanisms for metabolizing MeHg, any mechanism of elimination occurs through the placenta and a longer half-life in fetal than maternal blood was observed.

Therefore, as evidenced also by the Faroes study, the cord blood allows to assess the fetal exposure over longer time than maternal blood and appears to be the best biomarker for health risks assessment of the newborns [18,50,51]. Moreover, maternal blood sampling is invasive, requires proper equipment, and storage and transportation is more complicated.

Results from the Faroe Islands provided the basis for the National Research Council (NRC) and United States Environmental Protection Agency (USEPA) for defining a Reference Dose (RfD) for MeHg intake at 0.1 μg/kg bw/day, as an estimate of a daily exposure where no risk of harmful effects during a lifetime would occur [57]. This level corresponds to a Hg concentration of 5.8 μg/L in cord blood (assuming that MeHg accounts for ≥90%) and 3.5 μg/L in maternal blood [36,46].

Generally, hair’s Hg concentration is 250 to 300-fold higher than that in blood, because once transported into the follicular cells, the high content of cysteine residues of keratin proteins supplies the binding site for the transported MeHg [36]. Although inorganic and organic Hg are incorporated in hair structure, among fish consumers MeHg constitutes about 80% of the total Hg, and this matrix is widely used to estimate medium/long-term exposure (including fetal exposure) [46]. In addition, hair is an accessible (non-invasive) biological specimen, with cheap cost and easy to transport for laboratory analysis. However, reliable analytical results should be kept under control by quality assurance/quality control programs in laboratory [29]. In contrast to blood, hair concentration may integrate exposure over longer periods of time based on the following assumptions: (i) the blood Hg concentration is directly proportional to Hg found into the newly forming hair; and (ii) the growing hair shafts has similar rate and limited inter-individual variability. These assumptions imply the suitability of the maternal hair as biomarker of fetal exposure because hair Hg concentration is directly related to MeHg intake through the intermediary kinetic compartment of blood (Figure 1), as well a clear correlation between the portion of hair analyzed and the time of exposure. The common assumption of a growth rate of 1.1 cm per month for scalp hair indicates that approximatively 8 months of pregnancy could be covered by 9-cm of maternal hair [46]. Notwithstanding, several factors—such as ethnicity, age, gender, and color—may affect the hair-growth rate and MeHg incorporation, leading to temporal uncertainty and exposure misclassification [47,52]. Grandejan et al. [52] reported that the uncertainty of hair-growth rate became significant when a portion of hair equivalent to time period shorter than a single trimester were identified. Despite those limitations, several efforts have been carried out for determining guidance levels (e.g., benchmark doses, BMDs) based on hair Hg levels. The BMDs determined on the basis of the greatest follow-up studies (i.e., Seychelles, New Zealand and Faroes cohorts) indicated a level of 4–25 μg/g in maternal hair as a risk to infant [15,17,20,21,58,59]. Although a certain degree of uncertainty (e.g., shape of the dose–response curve, the choice of the cut-off as a benchmark response) makes that level currently under discussion, an increase of 1 µg/g in maternal hair and a reduction of 0.18 point in children’s intelligence quotient (IQ) is well established [60]. The safe daily reference dose set by USEPA (0.1 µg/kg bw/day) corresponds to a hair Hg concentration of 1 μg/g [46]; although updated calculation on developmental neurotoxicity at low-level exposure estimated a lower biological limit (0.58 μg/g) in hair [61].

A number of studies used Hg concentrations in nails as biomarkers of past exposure to MeHg, mainly in the context of nutritional epidemiology in children and cardiovascular effects of MeHg in adults [53,54,62]. A recent study suggested that, at early pregnancy, Hg in fingernails and toenails evidenced a maternal retroactive exposure of approximately 5 months whereas, at birth, a more recent MeHg exposure both for fetus and mother (i.e., especially during the third-trimester of gestation) can be assessed by means of these biomarkers [55]. An autopsy study on MeHg exposure assessment, suggested the measure of total Hg in toenails as a helpful complement to measurements of Hg in hair [63]. The advantages of nail Hg as biomarker are: non-invasive collection, cheap and easy to store and able to reveal potential chronic exposure [2]. In some studies, toenails were preferred due to less susceptibility to external contamination [54].

Umbilical cord tissue and breast milk were also used to determine fetal MeHg exposure levels [38,49,50,56,64,65,66]. The umbilical cord is formed in the early period of embryogenesis, but its full length is achieved when the second trimester ends, thus, assuming a biological half-life of 45 days for MeHg in tissues, umbilical cord-based biomarker may be useful to measure fetus exposure during the third trimester [29]. In the Faroes studies, the MeHg levels associated with neuropsychological deficits at 7 years of age were strongly predicted by Hg concentration both in cord tissue and cord blood, but less by weaker correlation between cord tissue and maternal hair [17]. The umbilical cord tissue offers advantages because it is easy to sample by noninvasive means, although it is not capable to identify short-term variations. Moreover, since the water content in the cord tissue varies substantially during the gestation, a dry weight-based Hg may be preferred over the wet weight-based level [50].

Mercury concentration in breast milk were used to assess the maternal past exposure, as well the potential exposure for breast-feeding infants. However, findings of decreasing inorganic Hg concentrations in maternal blood during breast-feeding indicate that it is readily excreted in breast milk and, then, MeHg-specific analysis in this matrix could be required [56].

### 3.2. Ethylmercury

Ethylmercury exposure occurs generally through thimerosal-containing vaccines (TCV) and due to intramuscularly injection its absorption is close to 100% at a very early age. Compared to MeHg exposure, EtHg exposure showed different kinetics in the human blood; it has been suggested that in children after thimerosal exposure (vaccination) the blood Hg half-life was from 3 to 7 days [5,67]. From infant blood EtHg is excreted more rapidly than MeHg, because, after injecting thimerosal, the fraction of EtHg converted to inorganic Hg helps the body to get rid of its toxic effects. Pioneering in vivo studies on the toxicokinetics of both organic species (EtHg and MeHg) reported high concentration of inorganic Hg from the dealkylation of EtHg in the kidneys suggesting a capacity to more easily reduce EtHg to Hg^2+^ [68]. Therefore, EtHg in blood is inherently less precise in estimating the internal dose [67]. Though some studies indicate that thimerosal-derived Hg can turn up in the infant hair as a result of chronic exposure [69] or acute exposure to TCV, in non-chronic condition the EtHg conversion to inorganic Hg could occur before it gets to the hair, making difficult the use of this matrix for a reliable assessment [70]. As a consequence, compared with MeHg, the problem in exposure assessment of EtHg could be related to its non-persistence in biological samples, making difficult the measurement of the internal dose and efforts to find a good biomarker in non-chronic conditions is still required.

### 3.3. Inorganic and Elemental Mercury

The major form of Hg in the urine is inorganic Hg and total Hg concentration is used to reflect the internal dose of the inorganic form [71]. Urine represents a suitable biomarker of long-term low-exposure to both inorganic and elemental Hg, because it contains Hg which accumulated in the renal tissue (i.e., kidney is the target organ) during a chronic exposure [72]; therefore, urinary Hg could be also a good indicator of body burden.

Inorganic Hg exposure could be preferably measured by using sample from a 24-h urinary collections. Excessive exposure may be considered for concentrations higher than 10–20 μg/L, above 100 μg/L neurological signs may be observed, but also at much lower levels (≤5–10 µg/L) they could take place [2]. However, if the Hg exposure is variable in intensity, Hg concentration in this matrix does not inevitably correlate with chronicity of toxic effects [73]. The usefulness of blood Hg level is related to its relatively short half-time as Hg returns to the background levels (below 5 μg/L) within few days after exposure; thus, this matrix may be used only after a short-term and high-level exposure to inorganic Hg [71].

Unlike inorganic Hg, elemental Hg is lipid soluble and can cross the blood-brain barrier. After absorption, it is rapidly converted to inorganic Hg and excreted in the urine, thus also a long-term elemental Hg exposure could be assessed by Hg concentration in this matrix [2]. Again, the blood level could be useful when measured soon after the exposure, because blood Hg levels peak sooner than urine levels. Mercury hair concentration is not a useful biomarker for both elemental and inorganic Hg due to the relatively minor amount of these species compared to organic Hg and the presence of external contamination [71]. Some studies examined the relationships between Hg concentrations in mammalian’s teeth following inhalation exposure of mercury vapor [74]. Since Hg deposits directly from the bloodstream into primary teeth during the mineralization process and enamel formation (that begins in utero and ends one year after birth), the spatial distribution of total Hg was used as an important tool in epidemiological studies relating to childhood development outcomes [75].

## 4. Health-Based Guidance Values for Hg

The public health risk-based values in terms of biomarker concentrations are available for several sources. These values correspond to biomarker concentrations consistent with exposure levels, previously deemed to be unlikely to result in adverse effects in the human population, including sensitive subgroups [76]. In Table 4 an overview of the published levels for Hg is shown. These levels are crucial for interpreting the HBM data (e.g., the statistical values of the population) and for promoting public health decisions or initiate policy measures, or both. They are based on epidemiological and toxicological studies, and population surveys.

The German HBM Commission defined two levels of health-related HBM values. The HBM I value corresponds to the Hg concentration of a substance in a human biological matrix below which no adverse health effects are expected. The HBM II value represents the concentration above which there is an increased risk of adverse health effects. The HBM-II is, thus, an intervention or action threshold level [77]. The health-based HBM-I and HBM-II values were 7 μg/L (or 5 μg/g creatinine) and 25 µg/L (or 20 μg/g creatinine) in urine and 5 μg/L and 15 μg/L in blood, respectively. No HBM values were set for hair by German HBM Commission [77].

The Joint Food and Agriculture Organization of the United Nations and WHO (FAO/WHO) Expert Committee on Food Additives (JECFA), based on the provisional tolerable weekly intake (PTWI) limit of 1.6 μg/kg bw/week for MeHg in order to protect fetus from neurotoxic effects, defined the 2.3 μg/g guidance value for Hg in hair [78]. The JECFA limit takes into in consideration the potential benefit of nutrients in fish (i.e., omega-3 fatty acids) against the MeHg toxicity.

The US EPA set a stricter RfD for chronic oral exposure to MeHg of 0.1 µg/kg bw/day for developmental neuropsychological impairment, that corresponds to the hair Hg concentration of 1 μg/g for children and women in reproductive age [46,79]. However, calculation from recent data on developmental neurotoxicity at exposure levels close to the background resulted in a biological limit in hair of*.* 50% below the recommended level (0.58 μg/g) [49,61,64,80,81]. From the RfD value—and assuming a ratio of MeHg in infant cord blood to maternal blood about 1.7:1.1 (e.g., 70% higher in cord than maternal blood) a maternal total Hg blood safe-concentration was set at 3.5 μg/L and in cord blood at 5.8 μg/L [36,46].

## 5. Cross-Sectional HBM Data

Several large cross-sectional HBM surveillance programs are conducted in the world and a summary of HBM data on total Hg and MeHg in neonates, infants, children and adolescents, and in pregnant women or women in child-bearing age is reported in Table 5.

The aims of the most national cross-sectional HBM strategy were to: (i) assess the Hg exposure of the population (either as screening-level exposure assessment of general population or in “hot-spot” exposure scenarios); (ii) provide reference values (RV_95_)—that is defined as the 95th percentile (P95) values selected from a representative cohort—to compare populations, subgroups or individuals; (iii) identify risk factors of exposure and highly exposed subject, and, in some cases; (iv) access samples in biobanks for future retrospective exposure assessment. Examples of national cross-sectional HBM surveys are those from the Czech Republic HBM program (Cz-HBM) [82,83], the German Environmental Survey (GerES) [84,85], Flemish Environment and Health Study (FLESH) [86], the French National Survey on Nutrition and Health (Etude Nationale Nutrition Santé, ENNS) [87], the Italian HBM survey [88,89], the US National Health and Nutrition Examination Survey (NHANES) [97], the Canadian Health Measures Survey (CHMS) [98]. Individual and smaller research studies, were also take into account in order to compare the Hg levels among different countries.

### 5.1. Mercury Level in Hair

Among the European populations significant variation in the hair total Hg level in children was depicted, with the highest Geometric Mean (GM) value in Spain (1.41 µg/L), Greece (1.36 µg/L), and Italy (median value: 0.78 µg/L), and France (0.37 µg/L), and approximately the same lower level (about 0.19 µg/L) in Belgium, Slovenia, and Czech Republic [35,83,86,87,89,91,92]. France survey conducted in 2006–2007 also indicated that some children present a P95 value (1.2 µg/L) above the 1.0 μg/g threshold level proposed by NRC [46,87]. The DEMOnstration of a study to COordinate and Perform Human biomonitoring on a European Scale (DEMOCOPHES) project (http://www.eu-hbm.info/democophes)—implemented in 17 European countries and aimed at assessing exposures to Hg enrolling 120 children (6–11 years) and their mothers (>45 years)—showed that the GM level of Hg hair in mothers was 0.23 μg/g and the P90 level was 1.20 μg/g, reflecting low to moderate fish consumption [96,106]. The DEMOCOPHES results showed also that younger children (6–8 years) were more exposed than older children (9–11 years of age), indicating the need to pay attention to younger children (data are not shown). For both, the Hg level were lower than the mothers, i.e., GM level of 0.15 μg/g and P90 level of 0.80 μg/g (Table 5). The final report (http://www.eu-hbm.info/euresult/media-corner/press-kit) explained that 1.4% of the children and 3.4% of the mothers presented hair Hg levels above the health-based guidance value of 2.3 μg/g set by FAO/WHO and 8.1% of the children and 12.7% of the mothers exceeded the 1.0 μg/g level proposed by NRC [46]. Bellanger et al. [61] analyzed the distribution of hair Hg concentrations among women in childbearing age originated from the DEMOCOPHES project and other literature data and determined, in each European country, the distribution of the population with Hg hair concentration above the health-based values proposed (0.58 µg/g; 1.0 µg/g; 2.3 µg/g) (see Table 4). The authors estimated that every year more than 1.8 million children were born with MeHg exposures above the 0.58 μg/g limit, 900,000 children were born from mothers whose level exceeds the 1.0 μg/g threshold level set by US EPA, and about 200,000 births exceeds a higher limit of 2.3 μg/g proposed by the FAO/WHO. Findings showed higher hair Hg concentrations in children of Southern European countries compared with those living in Northern Europe, and lowest exposure in the Eastern Europe. However, a recent study conducted by Višnjevec et al. [107]—which reviewed more than 50 studies published in Europe in the last ten years—indicated the lack of difference between Southern and northern European children populations. The latter study also revealed that the lowest Hg exposures in the central European countries and the highest values in children and mothers from coastal area, were directly related to local fish consumption. Also, studies on pregnant women and mothers of young children living in coastal areas of Italy and Greece found a MeHg hair concentration of approximately 1 µg/g or higher [89,91]. Other studies conducted in the Inuit population of Canada (GM: 3.7 µg/g), Faroe island (GM: 4.08 µg/g), Hong Kong (median value: 1.2 µg/g), and Japan (GM: 1.62 µg/g) arrived at the same conclusion [38,99,102,103]. However, the different conclusions reported in the reviews by Bellanger et al. [61], and Višnjevec et al. [107] may be the result of several factors like different sample size populations, lack of homogeneous method to assess fish intake, and oversimplification of the data [107].

### 5.2. Mercury Level in Blood

Blood Hg concentrations in children of the National European surveys were around 1 µg/L, with the highest central values found in Italy (GM: 0.84 µg/L), Slovenia (GM: 0.77 µg/L) and Sweden (median value: 1.1 µg/L), and lowest values in Czech Republic (GM: 0.45 µg/L) and Germany (GM: 0.23 µg/L) [83,85,88,92,93]. Moreover, also a smaller Swedish longitudinal study (98 neonates enrolled) showed that P95 value of MeHg in blood was above the USEPA’s threshold limit (3.5 µg/L) [94]. The levels found in these countries were also above the reference value (RV_95_) of 0.8 µg/L set for children (3–14 years) who ate fish ≤3 times per month by the German Human Biomonitoring Commission [77]. These coastal countries have banned or have restrictions or guidelines on amalgam in place [108]; therefore, the slightly higher exposure found in Italy, Slovenia and Swedish population could be due to higher fish consumption. In agreement, Višnjevec et al. [107] reported the lowest level of Hg in blood in general adult populations of central Europe, where also the lowest frequency of fish consumption was assessed. However, in none of European HBM populations survey the exposure levels exceeds the health-based HBM-I value of 5 µg/L. Differences in Hg blood levels were observed in adolescent between the periods of 2003–2004 and 2011–2012 in the US surveys (NHANES, see Table 5), where a slightly decrease in the newer survey was due to a lower Hg exposure from fish consumption. Species analyses of Hg in blood of general population enrolled during the NHANES in 2011–2012 showed values below to the detection limit for EtHg and inorganic Hg in all age groups, and in any case below to the levels related as a risk of exposure for MeHg [97]. Similar maternal blood Hg concentrations of the Inuit population of Canada (GM: 10.4 µg/L) to those found in Greenland (GM: 12.8 µg/L) confirmed that for both fish consumption was the main source of Hg [99,100]. These results are also in accordance with those obtained in the children population of South Korea and in pregnant women of Japan—who frequently consume fish—in which the highest central blood Hg value (GM: 1.73 µg/L and 5.18 µg/L, respectively) among the available surveys were depicted [38,101].

As for the Hg concentrations in blood, smaller research on Hg levels in cord blood showed lowest level in central Europe, ranged from 0.9 µg/L to 1.5 µg/L [107]. A total of 1883 mother and child pairs from a population-based cohort study in Spain during the period 2004–2008 showed higher cord blood (GM: 8.2 µg/L) than the current USEPA reference dose (5.8 µg/L for MeHg) [95], corroborating the highest level of Hg exposure in coastal population due to high fish consumption, as reviewed by Višnjevec et al. [107]. Similar values were found in communities with a high fish intake such as Canada Inuit population (GM: 18.5) [99], Greenland (GM: 25.3 µg/L) [100], Japan (GM: 9.8 µg/L) [38], Hong Kong (median: 8.8 µg/L) [103], Taiwan (GM: 9.2 µg/L) [104], and Polynesia (GM: 10.5 µg/L) [105].

### 5.3. Mercury Level in Urine

Regarding urinary Hg, level for Slovenian children populations (0.73 µg/g creatinine) was above the German Human Biomonitoring Commission’s RV_95_ (0.4 μg/L) set for children without amalgam fillings [92]. Slovenia, recently, closed one of the largest mine in Europe, and the amount of mining dregs containing high concentrations of Hg remained in the area may contribute to the high levels found. Geometric urinary mean in children enrolled during the older Germany survey in 1990–1992 GM: 0.54 μg/L) was higher than that in children enrolled in 2003–2006 (GM: <0.1 μg/L) [84,85]; the concomitant reduction of dental amalgam use in children could be the reason of this trend [109]. Children of Czech Republic also showed the same trend, with a GM values (0.45 µg/L) above the RV_95_ value during the period 2001–2003 [82], and a declining tendency in the newer monitoring period in 2008–2009 (0.26 µg/L) [83]. Values lower than the RV_95_ were observed for the US children population during the period 2007–2012 [97]. None of the HBM national surveys presented values exceeding the HBM-I health–based level of 7 µg/L.

Regarding smaller studies, Woods et al. [110] carried out an assessment of longitudinal exposure to Hg during a clinical trial, measuring the urinary Hg concentrations in 506 Portuguese children (8–18 years old). The study reported a clear association between urinary Hg and both the number of amalgam restorations and the time since placement, reaching in the amalgam group a peak (3.2 µg/L) at 2 years and then a decreasing to baseline levels (1.5 µg/L) by 7-year of follow-up. These results are also in agreement with those obtained in more recent longitudinal and cross-sectional research from Korea where the mean urinary Hg concentration in 463 children (8–11 years old) was 1.04–1.23 µg/g creatinine, from Mexico where the found mean urinary Hg concentration in 112 children (6–12 years old) was 2.10 µg/L, and in British children (6–10 years old) of 0.9–1.2 µg/g creatinine [111,112,113]. However, other studies showed that children without amalgam fillings and high fish intake excrete elevated amounts of Hg in urine demonstrating that also fish consumption may influence the urinary levels [114]. Analogously, a study conducted on 800 children living in three separate European regions from France, Poland and Czech Republic revealed that the urinary Hg concentrations in French children (GM about 0.9 µg/g creatinine), living near a lead and zinc smelter, did not differ from those living in the near not polluted areas. Comparison among urinary Hg in French children and that found in Poland (GM about 0.06 µg/g creatinine) or the Czech Republic (GM about 0.15 µg/g creatinine) suggested an increase due to the higher fish consumption in France rather than due to industrial pollution [115]. In a Swedish longitudinal study (about 256 pregnant women enrolled), median concentration of total Hg in urine at parturition was 1.6 µg/L that decreased during breast feeding, probably due to excretion into breast milk [94]. Data reviewed by Višnjevec et al. [107] showed a comparable geographical variation in urinary Hg concentrations among populations to those previously described for hair and blood. According to the authors, the lowest levels of urinary Hg observed in children living in central European countries, where dental amalgam is still in use [109], supports that this source is still important but less significant than fish intake [107].

## 6. Birth Cohort HBM Studies

HBM is generally a cross-sectional study (one time or over a short period sampling) that makes difficult the identification of spatial and temporal trends of the environmental contaminant. Especially for those chemicals, like Hg, where the exposure window of greatest sensitivity in utero could produce adverse health effects later in life, the evaluation of the lifelong exposure milieus is a basic concept for monitoring and assessing health risks. In the past 20 years, some of the national HBM cross-sectional surveys were complemented with longitudinal birth cohort studies that allowed to assess perinatal exposure (by biomarkers measured in specimens of the pregnant mother, in cord blood, or in breast milk) and, following up the children, to: (i) describe the degree of individual perinatal Hg exposure and the internal dose during pregnancy; (ii) monitor temporal and spatial patterns of exposure from birth; (iii) evaluate the health effects occurring on fetal and infant growth, and during childhood development; and (iv) link environmental factors and exposures to health, with the aim of informing and orienting public policy decision-making. An overview of existing cohorts and the data collected can be found at www.birthcohorts.net, a webpage that aims to highlight the available basic information (e.g., size of population, contaminants analyzed) on this topic. An overview of the main birth cohort studies set up across Europe as well as smaller longitudinal research is reported in Table 6.

An example is that of the French Longitudinal Study of Children “Etude Longitudinale Francaise depuis l’Enfance (ELFE)”; a national representative cohort of 20,000 children followed up through adulthood enrolled in 2009. The HBM study is ongoing and it is paying attention to interaction between Hg and other metals (aluminum, arsenic, cadmium, and lead), and the complexity of mixed exposures with several types of persistent organic pollutants [116]. Although analysis is still in progress, preliminary descriptive results for Hg indicate a decrease in exposure temporal trends [40]. The selection of a sub-cohort of 601 women (i.e., “Perturbateurs Endocriniens: Étude Longitudinale sur les Anomalies de la Grossesse, l’Infertilité et l’Enfance (PÉLAGIE)” cohort study) allowed to assess the annual economic benefits for the community as a function of the MeHg prenatal exposure reduction [32]. From pregnant women enrolled in the Avon Longitudinal Study of Parents and Children (ALSPAC) cohort in United Kingdom, Golding et al. [117] suggested that limiting seafood intake during pregnancy may have a limited impact on prenatal blood Hg levels.

A total of 4000 pregnant women engaged in different Spanish cohorts (1997–2008) allowed to verify the significant contribution of oily fish consumption on the high proportion of newborns with cord blood Hg concentration above the limit proposed by USEPA (5.8 μg/L for MeHg) [95]. About 100,000 pregnant women and children were recruited from 1996 to 2002 for a prospective population-based cohort study in Denmark (The Danish National Birth Cohort, DNBC) [118]. Findings from a sub-cohort of 25,446 subjects indicated independent association between the duration of breast feeding and maternal fish consumption with better childhood development, highlighting how fish is also a source of valuable nutrients and awarding the need to consider the benefit-risks balance from fish consumption [80]. In agreement, a sub-cohort of the Norwegian Mother and Child Cohort Study (MoBa)—in which more than 60,000 women were recruited—showed that a birth weight was positively associated with seafood intake in pregnancy and negatively with Hg exposure, emphasizing the needs to clarify at what level of Hg exposure this risk might exceed the benefits of seafood [119]. Other examples are those from Italy [89], Germany [120], Poland [64,121], Slovakia [122], Finland [123], and Greece [124]; measurements of Hg in some of them are planned and/or ongoing but not yet completed [126]. Although the general aims of the largest birth cohorts focused on Hg exposure were similar, their specific design and size could vary broadly. Moreover, many of them are ongoing under a multidisciplinary approach and focused also on the interactions and effects of combined neurotoxic compounds (e.g., multi-metals) and various endocrine disruptors [76], whilst the smaller cohorts are generally set to understand environmental risk factors and health outcomes in specific exposure context and communities. In this sense, facilitating exchange of knowledge and comparative analyses between the studies, and collaboration between cohorts and researchers, may be a general effort. At the EU level, the prospective Mediterranean cohort study (Northern Adriatic Cohort, NAC-II) included in the integrated project on the public health impact of long-term, low-level, mixed element exposure in susceptible population (PHIME: http://phime.oikon.hr/), enrolled approximately 1700 mother–infant pairs from 4 recruitment areas of Italy, Greece, Slovenia and Croatia. This approach paid attention to interaction between elements and the complexity of mixed exposures and allowed to establish a moderate but significantly beneficial effect of fish consumption—assigned to fatty acids in fish—in pregnancy on cognitive and language development in children from that European area [125]. This example clarified how the collaboration across large regions may improve the potential of birth cohorts and efforts toward a rational approach for sharing HBM data should be improved. In this way, other two EU projects are worthy of particular mention. The Cross-Mediterranean Environment and Health Network (CROME; www.crome-life.eu), project used an integrated methodology for interpreting HBM data of four European coastal countries (Greece, Italy, Slovenia and Spain). The CROME methodology linked the environmental monitoring, HBM data and epidemiological observation in order to define: (i) the variability of environmental exposure levels and other stressors (i.e., age windows, socioeconomic factors); (ii) the rational protocol able to assess the role of metal and neurodevelopment-related genetic polymorphisms. Partial results were recently published by Tratnik et al. [127].

The HEALS project (http://www.heals-eu.eu/) started in 2013 with the main objective of developing an integrated methodology for environment and health-based assessment by an “exposomic” approach, in which internal biomarkers of exposure are associated and integrated with measurements or modelling of exposures in air, soil, water, food, endogenous processes (hormones, oxidative stress, ageing) and genetic susceptibility, and other non-chemical stressors (individual social, economic and psychological environment) in order to characterize how these exposures relate to the development of health outcomes in groups of people.

## 7. Conclusions

HBM data, both from cross-sectional and longitudinal surveys, demonstrated that through this approach it is possible to identify subpopulation with elevated Hg exposures, and provide also the achievements of mitigating exposure at different levels (regional and even national). However, several factors as spatial distribution of Hg levels around the countries, laborious comparability of data (due to the lack of standardized approach in terms of population enrolled), sample collection, submission of food frequency questionnaires, and of supplement information (like lifestyle factors and consumer behavior), and data interpretation, restrict the global power of HBM activities for monitoring the effects of a global policy intervention—like the Minamata Convention. At the global scale exposure, new paradigms for a coordinated intervention are ongoing. The UNEP/WHO project is developing a plan for global monitoring of human exposure to Hg with the aims to: (i) harmonize approaches for monitoring Hg both in humans and environment; (ii) strengthen the capacity for Hg analysis to make accurate and comparable determinations. From our point of view, we believe that this is the unique and effective approach capable of: (i) characterize the actual exposure of the global population; (ii) verify the reductions in Hg human exposure after implementation of the Minamata treaty; (iii) understand the extent to which the perturbation of homeostatic pathways can lead to the development of adverse health outcomes; (iv) increase the number of HBM reference values and develop HBM health-based guidance values in a health risk assessment at global context. However, in order to support the evidence-based public health and environmental and biological measures, the great challenge for the next future as well as the further direction of HBM programs should also take into account the cumulative complexity of multiple exposures to chemicals, including new environmental stressors or emerging ones, and thereof interactions.

## Figures and Tables

**Table 1:** Major events for recognition of toxicity of methylmercury (MeHg) in children.

Year	Event	References
1952	First report on developmental MeHg neurotoxicity in infants	[8]
1956	Discovery of a seafood-related disease in Minamata Bay in Japan	[9]
1968	Acknowledgment of MeHg as cause of Minamata disease	[9]
1972	Experimental study on delayed effects of developmental neurotoxicity in rats	[11]
1973	Report on a dose-dependence of poisoning from methylmercury-derived fungicide by using Iraq data	[10]
1986	First epidemiology report on adverse effects in children related to maternal fish intake during pregnancy in New Zealand	[15]
1997	Confirmation from prospective study in the Faroe Islands on adverse effects in children from maternal fish intake during pregnancy	[17]
1995	The Seychelles Child Development Study was launched for evaluating prenatal methylmercury exposure resulting solely from ocean fish consumption	[19]

**Table 2:** Toxicological features of mercury (Hg) species.

Organic Mercury	Elemental Mercury (Hg^0^)	Inorganic Mercury (Mercurous Hg^+^; Mercuric Hg^2+^)
	*Source of exposure*	
- MeHg: diet (fish, rice) - EtHg: a topical antiseptic and antifungal agent in vaccines	- Hg vapours released from maternal dental amalgam (50% is Hg^0^) [41] - Accidental exposure (from broken thermometers and other devices used in school laboratories) - Specific products (e.g., mercury-containing paints)	Use of cosmetics containing salts (skin creams, soaps, medications); vaccines; breast milk for infants
	*Absorption*	
- Oral: MeHg from GI tract (95%) [4,29] - Transdermal: EtHg from vaccines (100%) [5] - Inhalation: only from vapours of MeHg	- Oral: metallic Hg in GI tract is converted to mercuric sulfide [4] - Dermal: absorption of Hg^0^ through children’s skin - Inhalation: from Hg vapours (70–85%) [2]	- Oral: absorption through the GI from breast milk (infants) or from water (children) - Dermal: absorption through children’s skin - Inhalation: aerosol from Hg salts
	*Distribution*	
- MeHg from the GI tract is distributed to the blood; in the body it is present as hydrophilic complexes attached to the sulfur atom of thiol ligands [46] - MeHg crosses the blood-brain or placental barrier via a MeHg-l-cysteine complex transported by neutral amino acid carrier [29,46] - After thimerosal injection, the EtHg–cysteine complex is exported from muscle cells by thiol-containing proteins. Then, it exchanges with generic plasma thiol proteins, like albumin [5]	After absorption it crosses the lungs and, thus, into the bloodstream, where, due to its high lipophilicity, is distributed throughout the body, including the blood-brain and the placenta barrier [7]	- From the GI tract it is distributed to the blood and organs. Mercuric Hg has affinity for sulfhydryl groups in the RBCs and plasma [4,47] - Due to its ionic charge it does not cross the blood-brain or the placenta barrier
	*Biotransformation (metabolism)*	
- MeHg is stable in the body, but intestinal flora, tissue macrophages, and fetal liver are site of demethylation to inorganic Hg [4] - EtHg is much more less stable; it is rapidly degraded to mercuric Hg	Elemental Hg is oxidized to mercuric Hg in the RBCs by catalase and hydrogen peroxide [4]	Mercuric Hg is unstable in vivo; it is converted to elemental Hg (rat study); only intestinal flora is site of methylation [46]
	*Excretion*	
- MeHg is secreted in bile and excreted in feces (~90% in feces occurs as inorganic Hg after demethylation) [46] - MeHg is excreted also in breast milk [2] - Approximately 1% of the human body burden of MeHg is excreted daily [2,46] - MeHg half-life elimination has been estimated at 45–90 days [4,46] - EtHg is excreted mainly by feces - From infant blood the EtHg excretion resulted more rapid than MeHg due to its rapid conversion to mercuric Hg (half-life 3–7 days) [5]	- Hg vapour is excreted via sweat and saliva, or as mercuric Hg via feces and urine - Approximately more than 1% of the human body burden is excreted daily [46] - Half-life elimination has been estimated at 58 days [4,46]	- Inorganic Hg is excreted mainly in urine; saliva, bile, sweat, exhalation, and breast milk are other routes of excretion - Half-life has been estimated at 49–96 days [4,46]
	*Target organs*	
MeHg and EtHg have the same target: fetal brain, CNS, other system (cardiovascular, reproductive, immune, etc.)	CNS, kidney, lungs, skin	Since mercuric Hg induces metallothionein production in the kidneys, the highest concentration is in this organ, however also CNS and skin are critical sites

MeHg: methyl-mercury; EtHg: ethyl-mercury; CNS: central nervous system; GI: gastrointestinal; RBCs: red blood cells.

**Table 3:** Characteristics and specific information on the MeHg biomarkers.

Biomarker	Exposure	Pros	Cons
Maternal blood	Short-term exposure	Total Hg is suitable for estimating internal exposure to MeHg (mother with regular fish consumption) [4] Total Hg in RBCs is a more accurate measure of MeHg exposure (procedure is more time-consuming) [47]	Invasive sampling Storage and transportation is more complicated Total Hg does not provide clear information about magnitude and timing of exposure [46]
Cord blood	Middle-term exposure	Total Hg reflects the MeHg concentrations in the target organ (i.e., fetal brain) [46] Total Hg represents fetal exposure over longer time than maternal blood [48]	Total Hg does not provide information on exposure variability during gestation [46] Storage and transportation is more complicated
Hair	Long-term exposure	Non-invasive sampling Easy to preserve Total Hg estimates internal mehg exposure at all exposure levels (fish consumer population) [46] Total Hg provides information about magnitude and timing of exposure [29]	Quality assurance/quality control systems are required for accurate results (presence of external contamination) [29] Uncertainties on the hair-growth rate [47,52]
Nails (finger- and toenails)	Long-term exposure	Simple and non-invasive sampling Easy to preserve Capable to reveal chronic exposure [53,54]	Quality assurance/quality control systems are required for accurate results [29,55] Fingernails are sometimes contaminated [54,55]
Umbilical cord tissue	Middle term exposure	Simple and non-invasive sampling Total Hg represents exposure during the third trimester [29]	Not capable to identify sensitive short-term variation [50] A dry weight-based total Hg concentration is more accurate (procedure is more time-consuming) [50]
Breast milk	Long-term exposure	Total Hg is suitable for estimating past maternal exposure Total Hg is suitable to predict the potential exposure for breast-feeding in infants [56]	MeHg-specific analysis may be required [56]

**Table 4:** Health-based guidance values for Hg in biological matrices.

	Reference Population	HBM-I [77]	HBM-II [77]	NRC, [46]	JECFA, [78]	Bellanger et al. [61]
Total Hg in urine	children and women of child-bearing age	7 µg/L (5 µ/g creat.)	25 µg/L (20 µ/g creat.)			
Total Hg in blood	children and women of child-bearing age	5 µg/L	15 µg/L			
MeHg in hair ^a^	children and women of child-bearing age			1 µg/g	2.3 µg/g	0.58 µg/g
Total Hg cord blood	-			5.8 µg/L		
Total Hg maternal blood	pregnant women			3.5 µg/L		

^a^ Dry weight; HBM: human biomonitoring; NRC: National Research Council; FAO: Food and Agriculture Organization of the United Nations; JECFA: Joint FAO/WHO Expert Committee on Food Additives; Creat.: creatinine.

**Table 5:** HBM data of the available National European Surveys and other large sample size population on total Hg and MeHg (when not specified, values are Geometric Mean (GM) and 95th percentile (P95)).

			MeHg	Total Hg				
Country	Study Period	Reference Population	Hair (µg/g)	Hair (µg/g)	Blood (µg/L)	Urine (µg/L)	Cord Blood (µg/L)	References
**Czech Republic**	Cz-HBM 2001–2003	Children (8–10 yo)			0.43 (1.44) *n* = 333	0.45 (4.18) ^a^ *n* = 619		[82]
	2008	Children (8–10 yo)		0.18 (0.61) *n* = 316	0.45 (1.39) *n* = 382	0.26 (2.19) ^a^ *n* = 364		[83]
**Germany**	GerES II 1990–1992	Children (6–17 yo)			0.33 (1.4) *n* = 812	0.54 (3.99) *n* = 812		[84]
	GerES IV 2003–2006	Children (3–14 yo)			0.23 (0.89) *n* = 1790	<0.1 (0.4) *n* = 1790		[85]
**Belgium (Flanders)**	FLEHS II 2007–2011	Mothers (20–40 yo)	0.26 (0.5) ^b^ *n* = 242	0.35 (0.60) ^b^ *n* = 242				[86]
		Adolescents (14–15 yo)	0.12 (0.35) ^b^ *n* = 206	0.19 (0.47) ^b^ *n* = 206				
**France**	ENNS 2006–2007	Children (3–17 yo)		0.37 (1.2) *n* = 1364				[87]
**Italy**	PROBE 2008–2010	Adolescents (13–15 yo)			0.84 (3.55) *n* = 252			[88]
	2007–2009	Pregnant women	1.38 ^c^ (1.85) ^e^ *n* = 220	0.78 ^c^ (1.28) ^e^ *n* = 604	0.0023 ^c^ (0.0039) ^e^ *n* = 606			[89]
**Austria**	2008–2010	Children (6–11 yo)	0.006 ^c^ *n* = 50					[90]
**Greece**		Pregnant women (17–46 yo)	1.07 *n* = 246	1.36 *n* = 246				[91]
**Slovenia**	Not available	women in childbearing age (20–35 yo)		0.24 *n* = 127	1.04 *n* = 127	0.73 ^a^ *n* = 127		[92]
		Children (6–11 yo)		0.18 *n* = 174	0.77 *n* = 174	0.73 ^a^ *n* = 174		
**Sweden**	1993–1994	Adolescent (15 yo)			1.1 ^c^ (2.7) *n* = 335			[93]
	1996–1999	Pregnant women (20–40 yo)		0.35 ^c^ (0.81) ^b^ *n* = 127			MeHg: 1.3 ^c^ (2.7) ^b^ In-Hg: 0.15 ^c^ (0.32) ^b^ *n* = 130	[42]
	1994–1996	Pregnant women (20–40 yo)			MeHg: 0.94 ^c^ (2.5) In-Hg: 0.37 ^c^ (1.4) *n* = 148	1.6 ^c^ (4.6) *n* = 226		[94]
		Newborns			MeHg: 1.4 ^c^ (3.8) In-Hg: 0.34 ^c^ (0.75) *n* = 98			
**Spain**	2008	Newborns and infants (0–4 yo)	0.97 *n* = 218	1.41 *n* = 218				[35]
	2004–2008	Mother and child pairs					8.2 *n* = 1883	[95]
**Poland**	2001–2003	Mother and child pairs			1.09 *n* = 233		0.8 *n* = 233	[64]
**17 EU countries**	DEMOCHOPES 2010–2012	Children (6–11 yo)		0.15 (0.80) ^b^ *n* = 120				[96]
		Mothers (<45 yo)		0.23 (1.20) ^b^ *n* = 120				
**USA—NHANES**	2003–2004	Infant (1–5 yo)			0.33 (1.8) *n* = 911			[97]
	2003–2004	Children (6–11 yo)			0.42 (1.95) *n* = 856	0.30 (1.87) ^d^ *n* = 398		
	2003–2004	Adolescent (12–19 yo)			0.49 (2.60) *n* = 2081	0.36 (1.82) ^d^ *n* = 375		
	2009–2010	Adolescent (12–19 yo)			0.53 (3.01) *n* = 1183			
	2011–2012	Infant (1–5 yo)			0.26 (0.99) *n* = 713			
	2011–2012	Children (6–11 yo)			0.33 (1.40) *n* = 1048	0.24 (1.37) *n* = 401		
					MeHg: 0.21 (1.34) *n* = 1044			
	2011–2012	Adolescent (12–19 yo)			0.41 (2.25) *n* = 1129	0.26 (1.31) *n* = 390		
					MeHg: 0.27 (2.15) *n* = 1121			
**Canada**	CHMS 2009–2011	Infant (1–5 yo)			0.27 (3.0) *n* = 495			[98]
	CHMS 2009–2011	Children (6–11 yo)			0.28 (2.0) *n* = 961			
	CHMS 2009–2011	Adolescent (12–19 yo)			0.27 (2.4) *n* = 997			
	Inuit popluation 1995–2001	Pregnant women (14–40 yo)		3.7 *n* = 130	10.4 *n* = 130		18.5 *n* = 130	[99]
**Greenland**	1994–1996	Pregnant women			12.8 *n* = 180		25.3 *n* = 178	[100]
**South Korea**	KorEHS-C 2011–2012	Children and adolescent (6–19 yo)			1.73 (3.20) *n* = 351			[101]
**Faeroe Island**	1994–1995	Mothers (20–35 yo)		4.08 *n* = 144				[102]
**Hong Kong**	2000–2001	Mother and child pairs		1.2 ^c^ *n* = 1057			8.8 ^c^ *n* = 1057	[103]
**Taiwan**	2004–2005	Pregnant women (16–42 yo)					9.2 *n* = 65	[104]
**Japan**	1996	Pregnant women (19–41 yo)		1.62 (2.19) ^e^ *n* = 116	5.18 (7.34) ^e^ *n* = 116		9.8 (13.6) ^e^ *n* = 116	[38]
**Polynesia**	2005–2006	Pregnant women (15–44 yo)					10.5 (11.5) *n* = 242	[105]

yo: years old; ^a^ urine in µg/g creatinine; ^b^ value in brackets is 90th percentile; ^c^ median value; ^d^ data of NHANES 2007–2008; ^e^ value in brackets is 75th percentile. Cz-HBM: Czech-Human biomonitoring; GerES: German Environmental Survey; FLEHS: Flemish Environment and Health Study; ENNS: Étude nationale nutrition santé (French: National Nutrition and Health Survey); PROBE: PROgramma per il Biomonitoraggio dell’Esposizione della popolazione generale (Italian Programme for biomonitoring the general population exposure); DEMOCHOPES: DEMOnstration of a study to COordinate and Perform Human biomonitoring on a European Scale; NHANES: National Health and Nutrition Examination Survey, United States of America; CHMS: Canadian Health Measures Survey; KorEHS: Korean Environmental Health Survey.

**Table 6:** Overview of European birth national cohorts.

Country	Birth Cohort	Metals	Enrollment Period	No. of Children at Birth	References
Faroe Islands	Faroes: Children’s Health and the Environment in the Faroes	Hg, Pb, Se	1986–2009	2351	[17,45]
United Kingdom	ALSPAC—The Avon Longitudinal Study of Parents and Children	As, Cd, Hg, Mn, Pb, Se	1991–1992	14,062	[117]
Denmark	DNBC—Danish National Birth Cohort	Hg	1996–2002	96,986	[118]
Spain	INMA—Environment and Childhood	Hg, Pb, TMS	1997–2008	3757	[95]
Norway	MoBa—Norwegian Mother and Child Cohort Study	Hg	1999–2008	100,000	[119]
Germany	Duisburg cohort	Cd, Hg, Pb, Se	2000–2003	234	[120]
Poland	Kraków cohort	Cd, Hg, Pb	2000–2003	505	[64]
	REPRO_PL—Polish Mother and Child Cohort	Cd, Hg, Pb, Se, Zn, Cu	2007–2011	1800	[121]
Slovakia	PCB cohort—Early Childhood Development and PCB exposures in Slovakia	Hg, Pb	2001–2003	1134	[122]
Finland	LUKAS cohort: Finnish cohort	As, Cd, Hg, Pb, Se	2002–2005	442	[123]
France	PÉLAGIE—Endocrine disruptors: longitudinal study on pregnancy abnormalities, infertility, and childhood	Hg	2002–2006	3421	[32]
	ELFE: French longitudinal study of children	Al, As, Cd, Hg, Pb	2011–2012	20,000	[116]
Italy	Trieste Cohort: Trieste child development cohort	Hg, Pb, Se, Zn	2007–2009	900	[89]
Greece	RHEA—Mother Child Cohort in Crete	As, Cd, Hg, Mn, Pb	2007–2008	1500	[124]
Italy, Greece, Slovenia, and Croatia	NACII—Mediterranean cohort study, (within PHIME project)	Cd, Hg, Pb, Mn, Se, Zn	2006–2011	1700	[125]

INMA: INfancia y Medio Ambiente (Spanish: Environment and Childhood); REPRO_PL: Polish Mother and Child Cohort; PCB: polychlorinated biphenyl; PÉLAGIE: Perturbateurs Endocriniens: Étude Longitudinale sur les Anomalies de la Grossesse, l’Infertilité et l’Enfance (French: Endocrine Disruptors: Longitudinal Study on Disorders of Pregnancy, Infertility and Children; ELFE: Etude Longitudinale Francaise depuis l’Enfance (French Longitudinal Study of Children); NACII: Northern Adriatic Cohort; PHIME: Public Health Impact of long-term low-level Mixed Element Exposure.
